# Patient-Oriented Priorities for Pediatric Erythromelalgia: A Priority-Setting Process

**DOI:** 10.3390/children12111477

**Published:** 2025-11-02

**Authors:** Don Daniel Ocay, Meghan Halpin, Ella Ford, Karen Keighley, Neva Keighley, Nikki Ramsay, Tayla Ramsay, Camelia M. Sheridan, Sarah M. Sheridan, Kirsten R. Tice, Deirdre De Ranieri, See Wan Tham, Catherine A. Brownstein, Jacqui Clinch, Dawn Marie Davis, Carolina Donado, Genevieve D’Souza, Deepa Kattail, Kimberly Lobo, Danielle Ravetti, Paola Sandroni, Jennifer N. Stinson, Gary A. Walco, Suellen M. Walker, Timothy W. Yu, Charles B. Berde

**Affiliations:** 1 Department of Anesthesiology, Critical Care and Pain Medicine, Boston Children’s Hospital, Boston, MA 02115, USA; 2 Department of Anaesthesia, Harvard Medical School, Boston, MA 02115, USA; 3 Patient/Parent Partner; 4 Division of Rheumatology, Ann & Robert H. Lurie Children’s Hospital of Chicago, Chicago, IL 60611, USA; 5 Department of Pediatrics, Feinberg School of Medicine, Northwestern University, Chicago, IL 60611, USA; 6 Department of Anesthesiology and Pain Medicine, School of Medicine, University of Washington, Seattle, WA 98105, USA; 7 Department of Anesthesiology and Pain Medicine, Seattle Children’s Hospital, Seattle, WA 98105, USA; 8 Division of Genetics and Genomics, Department of Pediatrics, Boston Children’s Hospital, Boston, MA 02115, USA; 9 Department of Pediatrics, Harvard Medical School, Boston, MA 02115, USA; 10 Department of Pediatric Rheumatology, Bristol Royal Hospital for Children, Bristol BS2 8BJ, UK; 11 Department of Dermatology, Mayo Clinic Rochester, Rochester, MN 55905, USA; 12 Department of Pediatric and Adolescent Medicine, Mayo Clinic Rochester, Rochester, MN 55905, USA; 13 Department of Anesthesiology, Perioperative and Pain Medicine, Stanford University, Stanford, CA 94305, USA; 14 Department of Anesthesia and Pain Medicine, The Hospital for Sick Children (SickKids), Toronto, ON M5G 1X8, Canada; 15 Department of Neurology, Mayo Clinic Rochester, Rochester, MN 55905, USA; 16 Research Institute, The Hospital for Sick Children (SickKids), Toronto, ON M5G 1X8, Canada; 17 Developmental Neurosciences, University College London Great Ormond Street Institute of Child Health, London WC1N 1EH, UK; 18 Department of Paediatric Anaesthesia and Pain Medicine, Great Ormond Street Hospital NHS Foundation Trust, London WC1N 3BH, UK

**Keywords:** pediatric erythromelalgia, priorities, advocacy, patient-oriented

## Abstract

**Background/Objectives**: Erythromelalgia is a rare condition characterized by burning pain, redness, and warmth primarily in the extremities, usually worsened by heat and alleviated by cold. The objective of this study was to identify the top 10 priorities in pediatric erythromelalgia from multiple perspectives, including clinicians, people with lived experience of childhood-onset erythromelalgia, and their family members. **Methods**: A modified James Lind Alliance Priority-Setting Process was conducted. The top priorities were identified through four phases: (1) an international online survey to gather priorities, (2) data processing, (3) an interim prioritization online survey, and (4) a virtual workshop to set the final priorities. **Results**: In phase 1, 185 potential priorities were submitted by 74 respondents (53% patients, 24% family members, and 23% clinicians) that were developed into 68 unique research questions (phase 2). In phase 3, of the 68 questions, 50 were rated for importance by 58 participants (38% patients, 36% family members, and 26% clinicians), reducing the list to 25 questions. In phase 4, the top 10 was reached through consensus by 12 participants (33% patients, 25% family members, and 42% clinicians) across Canada, South Africa, the United States of America, and the United Kingdom. **Conclusions**: The final priorities focused on the treatment of erythromelalgia, understanding underlying mechanisms, the association of erythromelalgia with various body systems, and generating awareness. This list is the first international patient-centered research agenda for childhood-onset erythromelalgia and a call to action from key partners to improve future research and care.

## 1. Introduction

Erythromelalgia is a rare condition characterized by burning pain, redness, and warmth primarily in the hands and feet [1]. Symptoms are usually worsened by heat and exercise and alleviated by cold, rest, and elevation. Pediatric erythromelalgia can be categorized as primary inherited (i.e., the presence of family history with or without genetic confirmation), primary symptomatic (i.e., no family history), or secondary erythromelalgia (i.e., associated with another disease). Our team conducted a scoping review [1] of the literature on childhood-onset erythromelalgia and observed significant heterogeneity in how healthcare providers reported symptoms, described the condition, and decided on the criteria for diagnosing pediatric erythromelalgia. Furthermore, there was a lack of evidence to guide the management of pediatric erythromelalgia, and the current treatment largely involves a stepwise trial-and-error approach.

Patient engagement is a priority and has evolved as standard practice in research. Strong community partnerships throughout the research cycle can lead to enhanced quality and impact of research efforts and consequently improved health outcomes. Marzban et al. highlight that patient engagement can improve treatment outcomes, patient compliance, and self-efficiency [2]. Given the limited availability of funds for research on rare diseases, priority-setting processes may help determine how to allocate those resources [3]. Our team has developed a multicenter PEDiatric ErythoMElalgia Registry Gathering multidisciplinary Experts (PED-EMERGE). It was important to ensure that the design, operations, and evaluation of evidence from the registry represented the perspectives of key partners, and the research addressed relevant priorities and led to useful and actionable findings. Therefore, an initial step to creating this PED-EMERGE and a primary objective of this study was to identify priorities on pediatric erythromelalgia from multiple key partners, including clinicians, people with lived experience (PWLE) of childhood-onset erythromelalgia, and their family members. This builds on our scoping review by identifying and addressing the needs from key partners, instead of identifying gaps in the scientific literature. The goals of this priority-setting process (PSP) were to (1) collaborate with patients, caregivers, and clinicians to identify knowledge gaps in pediatric erythromelalgia, (2) agree through consensus on a prioritized list of research questions, and (3) publish the results of the PSP.

## 2. Materials and Methods

### 2.1. Priority-Setting Process

The James Lind Alliance PSP brings patients, caregivers, and clinicians together to identify and prioritize the evidence uncertainties, or ‘unanswered questions’, that they agree are the most important for research in a specific topic area [4]. The James Lind Alliance PSP’s methodology is recognized as robust, strategic, objectively based, and inclusive and as promoting equity in patient voices [5]. The top priorities were identified through 4 phases: an international survey to gather priorities (phase 1), data processing (phase 2), interim prioritization (phase 3), and a workshop to set the final priorities (phase 4).

The steering committee overseeing the PSP included global representation of 1 patient partner (D.R.) and 12 multidisciplinary clinicians and/or researchers (D.D.O., C.B.B., C.A.B., J.C., D.M.D., G.D., D.K., K.L., P.S., J.N.S., G.A.W., and S.M.W.) with experience in caring for or conducting research on youth with erythromelalgia or a PSP [6,7,8]. Additional patient and parent partners (K.K., N.K., N.R., C.S., S.S., and two who wished to remain anonymous) were involved particularly in phase 2. This collaborative initiative enhanced the interpretability, implementation, credibility, and reach of the PSP phases and results (Table 1). Although the James Lind Alliance PSP is conceptualized as service evaluation and development, upon consultation with the Boston Children’s Hospital Research Ethics Board, ethics approval was obtained in March 2024 for all phases (IRB#: P00047307).

### 2.2. Phase 1: International Survey

An international survey on REDCap [9,10] gathered priorities from those with lived experience with pediatric erythromelalgia (current children above the age of 8 years old or adults), family members or caregivers of those with lived experience with pediatric erythromelalgia, and clinicians who treat children with erythromelalgia. A Multi-Language Management module in REDCap gave the ability to translate all text within REDCap. Respondents described up to five priorities in response to open-ended questions, and demographic information (age, gender, ethnicity, geographic location, healthcare profession, etc.) was collected. The survey was promoted via social media (X and Facebook), at pediatric pain programs and other pediatric centers (emails), through professional and patient organizations (newsletters, blog posts, emails), and via targeted emails to pediatric clinicians. Consent was provided upon completion of the anonymous survey. No incentive was offered for survey completion.

### 2.3. Phase 2: Data Processing

Out-of-scope submissions (i.e., not about pediatric erythromelalgia) were removed. Single survey responses with multiple research questions were separated. Eligible responses were categorized based on broad emerging themes to avoid duplication and combine related research questions. When possible, original responses were rewritten into researchable questions using the PICO format (Patient or Population, Intervention, Comparator or Control, and Outcome). For example, a response such as “managing pain” would be rewritten as “What treatments or strategies are the most effective for treating pain in youth with erythromelalgia?”. To reduce the number of research questions to fewer than 70, we retained questions that were derived from at least 2 unique survey respondents, at least 1 of whom had lived experience with pediatric erythromelalgia.

All research questions were verified as a true uncertainty, upon consulting our team’s scoping review [1] of the literature on pediatric erythromelalgia, before they went forward for prioritization in phase 3.

### 2.4. Phase 3: Interim Prioritization

We used an interim online survey on REDCap to reduce the list of research questions to 25–30 questions to be discussed at the priority-setting workshop. Participants were asked to choose their top 10 questions from the list of questions derived from phase 2 that they thought were the most important for researchers to answer. Demographic information was collected like in phase 1. The survey was sent through targeted invitations to participants from phase 1 who had provided their email address and agreed to be contacted for the subsequent phases and was promoted via social media (X, Facebook), at pediatric pain programs and other pediatric centers (emails), through professional and patient organizations (newsletters, blog posts, emails), and via targeted emails to clinicians in pediatric practice. Consent was provided upon completion of the anonymous survey. No incentive was offered for survey completion. We identified the 25 most highly selected research questions from each of the three partner groups (PWLE, family members, and clinicians). In circumstances where the interim prioritization did not produce a clear ranking or cut-off point (i.e., 25–30 questions), the steering committee decided which questions were taken forward to the final prioritization, with priority given to questions that were chosen by at least 2 groups.

### 2.5. Phase 4: Priority-Setting Workshop

The workshop to set the final priorities was held over a virtual platform (Zoom) in English. The goal of this workshop was to reach consensus on the final top 10 priorities selected from the 25–30 most important questions identified in the interim prioritization. Potential participants had been involved in prior phases and agreed to be contacted or were identified by the steering committee’s networks and partner organizations. A screening and demographic questionnaire was completed by interested participants to assess equal representation from patient, family, and clinician partner groups, as well as diversity in important areas identified by the steering committee (e.g., age, gender, ethnicity, geographic location, healthcare profession, etc.). Participants gave informed consent or assent prior to the workshop. Prior to the workshop, all participants chose and ranked their top 10 research questions from the reduced list. The workshop used a nominal group technique with small-group discussion and rankings (90 min), followed by whole-group review (30 min), a second round of small-group discussion and rankings (60 min), and a final whole-group review to reach consensus (60 min). Round-robin voting was used, with a clear majority (≥75%) required to reflect the consensus of the participants.

The James Lind Alliance recommends obtaining feedback after the final workshop to help understand how the process worked for each participant and address any individual concerns. At the end of the workshop, all participants completed the Public and Patient Engagement Evaluation Tool version 2.0 [11,12]. The tool included 13 statements rated on a 5-point Likert scale from 1 (“strongly disagree”) to 5 (“strongly agree”) and 6 open-ended questions assessing key elements of quality public and patient engagement: (1) integrity of design and process, (2) influence and impact, (3) participatory culture, and (4) collaboration and common purpose.

## 3. Results

**Figure 1:**
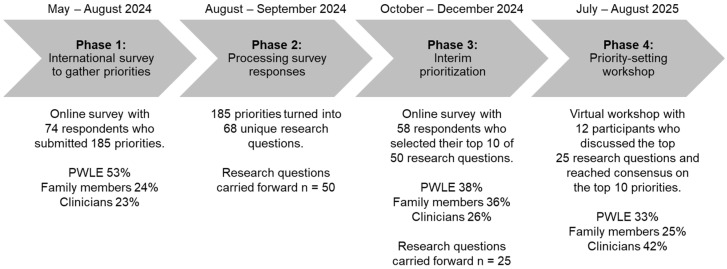
An overview of the priority-setting process for pediatric erythromelalgia.

Figure 1 provides an overview of results from the PSP. Demographic characteristics for all participant phases are reported in Table 2.

### 3.1. Phase 1: International Survey

The survey was viewed 143 times and was completed by 74 participants (completion rate of 52%), which included 39 PWLE (53%), 18 family members (24%), and 17 clinicians (23%). A total of 185 research priorities were raised, with a median of 2 priorities (range 1–6) per participant.

### 3.2. Phase 2: Data Processing

Of the 185 survey responses, one was excluded for being a personal story/anecdote. The remaining responses were combined into 68 research questions. Upon further verification, 18 questions were not identified to be true uncertainties (Supplementary Table S1) [13,14,15,16,17]. These questions were regarding access to genetic screening or clinical trials, the latest findings regarding the role of the *SCN9A* gene in erythromelalgia, available educational resources, etc. Therefore, 50 research questions were carried forward for interim prioritization.

### 3.3. Phase 3: Interim Prioritization

The interim prioritization survey was viewed 91 times and was completed by 58 participants (completion rate of 52%), which included 22 PWLE (38%), 21 family members (36%), and 15 clinicians (26%). Variability in the 25 most highly selected research questions from each of the three groups was observed (Supplementary Table S2). For example, PWLE highly selected 1 question on access, 2 on genetics, 5 on support, 7 on knowledge of erythromelalgia, 1 on physical activity, 1 on quality of life, 1 on school, 1 on sleep, 1 on transition to adulthood, and 8 on treatment. On the other hand, clinicians highly selected 1 question on access, 2 on genetics, 1 on support, 7 on knowledge of erythromelalgia, 2 on physical activity, 1 on quality of life, 2 on transition to adulthood, and 10 on treatment. Upon aggregating the responses from each group, and consultation with the steering committee, 25 questions moved forward to the final prioritization workshop.

### 3.4. Phase 4: Priority-Setting Workshop

Twelve participants (four PWLE [33%], three family members [25%], and five clinicians [42%]) attended the workshop to discuss the top 25 questions from phase 3. The final workshop was split into two parts to accommodate various time zones. The first part consisted of the two small-group discussions and rankings held on separate days so participants could choose the session best suited to their availability and international time-zone. Upon merging the rankings from both groups, and the high overlap in rankings, the second part consisted of a final whole-group review (3 PWLE [30%], 3 family members [30%], and 4 clinicians [40%]) to reach consensus. One PWLE and one clinician were unable to attend the second part but gave feedback on the merged responses from both groups prior to the whole-group review. The final top 10 list is presented in Box 1. Although the list was ranked, participants acknowledged that the research questions can be answered in parallel, and the rankings do not necessarily reflect that one priority is more important than another.

Workshop participant engagement evaluations are presented in Supplementary Table S3. Evaluations from the final workshop revealed successful engagement with the different partner groups. Participants reported that the collaboration between the different groups created a safe and respectful environment, validated erythromelalgia as a rare condition, and created a sense of hope for the future. Comments from the open-ended questions centered around community engagement and acknowledged the clear coordination of the process. It was evident that effective communication strategies were key to keeping participants engaged, despite the virtual format. Future considerations highlighted by the participants were to provide more background information on the research process.

Box 1 **Top 10 patient-oriented research priorities in pediatric erythromelalgia.**

1. Which medical therapies are the most effective for treating symptoms, particularly pain, in youth with erythromelalgia, dependent on the different (pheno)types of erythromelalgia?
2. What are the pathophysiological mechanisms, including genetics, underlying pediatric erythromelalgia?
3. What is the association between erythromelalgia and intrinsic/extrinsic triggering events (surgery, trauma, hormones, puberty, menarche, menstruation, pregnancy, menopause, immune, inflammatory, vascular)?
4. What strategies are effective for maintaining a good quality of life (e.g., mental health, participating in activities, sleep quality, good peer relationships, selfesteem, adaptations in school/work environments, etc.) in youth with erythromelalgia?
5. Which complementary nonpharmacological strategies (e.g., physical therapy, psychological therapy, lifestyle adaptations) are effective for youth with erythromelalgia?
6. Which approaches are the most effective for identifying and preventing flareups (e.g., during physical activity or sleep) in youth with erythromelalgia?
7. Which strategies (e.g., continued education, flyers, perspective from people with lived experience) are the most effective to educate the medical community and public (e.g., school, work, etc.) about erythromelalgia?
8. How can youth with erythromelalgia access multidisciplinary experts (including pain physicians, neurologists, rheumatologists, dermatologists, etc.) for diagnosis, management, and care coordination?
9. What are the different (pheno)types of erythromelalgia and their prognosis?
10. What is the longterm effect of erythromelalgia in youth (“effect” refers to the result of having erythromelalgia during childhood, as an adult in the future)?

## 4. Discussion

Through the process of priority-setting from an international collaboration with people with lived experience, their family members, and clinicians, we identified the top ten priorities for pediatric erythromelalgia. Similarly to other PSPs for rare diseases [3], these addressed broad themes including knowledge about erythromelalgia, potential interventions, assessment, awareness of the condition, and access to care, with an overall goal to improve quality of life. Half of the priorities (#1, 4, 5, 6, 8) focused on the treatment of erythromelalgia, with a particular emphasis on pain, a key symptom of the condition. These priorities also emphasize the need for additional research to better understand the underlying mechanisms and triggering events of childhood-onset erythromelalgia, as well as preventing the flare ups that emphasize the episodic pattern of symptoms in erythromelalgia (#2, 3, 9). Patients with erythromelalgia are managed by a range of specialists, such as pain medicine physicians, geneticists, dermatologists, neurologists, and rheumatologists, but this can vary and be dependent on local expertise and availability. Moreover, there are no guidelines to the treatment of erythromelalgia. As a result, the clinicians involved, and the priorities identified in this project, reflect a multidisciplinary approach incorporating multimodal (pharmacological and non-pharmacological) management of pediatric erythromelalgia.

Similarly to other priority-setting processes for pediatric [6] and adult [18] pain research, quality of life (#4) and complementary non-pharmacological approaches (#5) were highlighted as priorities. While some case studies and case series have reported on positive outcomes from non-pharmacological approaches [1], the quantity and quality of evidence are low, and factors contributing to quality of life, such as mental health, physical function, school attendance, and sleep quality, are not commonly reported. Understanding the pathophysiology of pediatric erythromelalgia and identifying phenotypes will offer an initial step to determining individualized therapeutic approaches. Moreover, the potential interactions between the nervous, vascular, endocrine and immune systems may give further insight into the neurobiology of pain that may be applicable to more common chronic pain conditions.

Two priorities related to increasing awareness in healthcare providers and the general public about childhood-onset erythromelalgia to improve support (#7, 8), while another two focused on the long-term effects of erythromelalgia as youth with erythromelalgia transition into adulthood (#9, 10). Multiple studies have shown that childhood-onset erythromelalgia impacts mental health and can increase depression and anxiety symptoms [1]. The need for increased awareness by the general public was highlighted in the final workshop, as the provision of adjustments in the school or work environment (e.g., control of environmental temperature and factors that trigger flares) may be difficult. Pain is sometimes dismissed and considered an invisible disability. Broad advocacy with increased public awareness is warranted to address the direct and indirect costs associated with chronic pain associated with erythromelalgia, especially as youth transition to becoming active members of society as an adult. The need for increased education for healthcare providers was also highlighted. Systems-level change educating healthcare providers on rare conditions, such as erythromelalgia, is warranted to provide safe, empathic, and effective patient-centered care.

Certain limitations were present for this study. First, although a deep inclusion model was employed to include diversity in every process, there was possible underrepresentation in race and gender, particularly in the final workshop. Moreover, there was a large representation of participants from the United States of America, and the final workshop was conducted in English. Although in our scoping review, when race or ethnicity was reported, cases predominantly occurred in White patients, cases have been reported across North America, Europe, Asia, and South America. The inclusion of non-English speakers into the final workshop may have representatively reflected key partners on childhood-onset erythromelalgia. However, for a rare condition, the final list represented various domains related to erythromelalgia that were consistently carried forward from phase 1 of the PSP. For example, one of the priorities (#3) includes certain milestones, such as menarche, menstruation, pregnancy, and menopause, but also includes additional triggering events such as surgery, infection, trauma or inflammation that may be experienced by females or males with erythromelalgia. A future priority-setting process may include conducting phase 1 of the PSP at patient-involved conferences/meetings as well or having live translators for the final workshop. Second, while most patients received care from primary care providers (family doctors/pediatrician), they were underrepresented in the final workshop. However, those who participated were clinicians from diverse subspecialties (e.g., rheumatology, dermatology, pain medicine, neurology) whose clinical practice was composed of youth with erythromelalgia.

The final priorities are a call to action from key partners on childhood-onset erythromelalgia. The list provided insight into priority common data elements across multiple centers for the PED-EMERGE collaboration. There has been momentum towards funding for rare diseases, with federal and non-federal opportunities, requesting applications year-round, due to the larger implications rare diseases may have for the general population (e.g., the development of non-opioid analgesics [19]). Examples from the United States of America include opportunities from the National Health Institute and Food and Drug Administration (e.g., RFA-FD-25–017) and from the Patient-Centered Outcomes Research Institute (pcori.org). This priority-setting initiative should be used to advocate for patient-oriented comparative clinical effectiveness research funding for this rare condition due to its severity rather than its prevalence [20]. Although this PSP focuses on erythromelalgia, other rare diseases collectively share substantial health and social care needs, and it is not that the system neglects rare diseases but that it neglects severe diseases that happen to be rare. The advancement of research into pediatric erythromelalgia will require creating new but also maintaining and strengthening current collaborations with multiple partners, including those involved in this PSP, as well as other researchers, healthcare providers, and the public. This list does not direct how or the order in which these questions should be investigated. Collaborations between basic, translational, and clinical science are warranted, with consideration of including industry partners, conducting implementation research, and continuous public engagement to increase the quantity and quality of evidence to guide treatment and management. Future work should focus on implementing these priorities in multicenter research collaborations, exploring cost-effectiveness, and ensuring equitable access for patients across different health systems.

## Figures and Tables

**Table 1:** Phases of the priority-setting process and steering committee activities [6].

Phase	Description	Activities
1: International survey (7 May 2024–10 August 2024)	An online open survey was used to gather potential priorities with a convenience sample of people with lived experience with pediatric erythromelalgia, family members/caregivers, and healthcare providers. Survey data were collected and managed with REDCap electronic data-capture tools hosted at Boston Children’s Hospital. The survey was open for about 3 months. Submissions were screened for duplicate entries based on priority responses, but none were found.	The survey questions were developed by the project lead (D.D.O.) and reviewed by the multidisciplinary steering committee members (C.A.B., J.C., D.M.D., G.D., D.K., K.L., D.R., P.S., J.N.S., G.A.W., S.M.W., C.B.B.). The survey promotional materials were reviewed and disseminated by the project lead (D.D.O.) to targeted patient organizations (The Erythromelalgia Association (burningfeet.org), and The Erythromelalgia Warriors (erythromelalgiawarriors.ning.com)) and pediatric pain programs (e.g., Pain in Child Health) and healthcare providers.
2: Data processing (August–September 2024)	The survey submissions were turned into unique testable research questions.	The project lead (D.D.O.) processed the responses, which were then reviewed by the multidisciplinary steering committee members (C.A.B., J.C., D.M.D., G.D., D.K., K.L., D.R., P.S., J.N.S., G.A.W., S.M.W., C.B.B.) followed by patient and parent partners (K.K., N.K., N.R., C.S., S.S., and two who wished to remain anonymous) for relevance, accuracy, clarity of wording, and duplication.
3: Interim prioritization (21 October 2024–23 December 2024)	An online open survey was used to identify 25–30 of the most important questions from a convenience sample of people with lived experience of pediatric erythromelalgia, family members/caregivers, and healthcare providers. Survey data were collected and managed as in phase 1. The survey was open for about 2 months. Previous respondents from phase 1 were given unique survey links to avoid duplicate entries.	Patient and parent partners (D.R., K.K., N.K., N.R., C.S., S.S., and two who wished to remain anonymous) selected the “choose 10” method for ranking the importance of each research questions.
4: Priority-setting workshop (Part 1 Group 1: 9 July 2025; Part 1 Group 2: 24 July 2025; Part 2: 15 August 2025)	Two-part virtual workshop to reach consensus on the final top 10 list of research priorities with a representative group of participants with lived experience of pediatric erythromelalgia, family members/caregivers, and healthcare providers.	The project lead (D.D.O.) identified important areas of representation to ensure diversity of workshop participants with input from the multidisciplinary steering committee members (C.A.B., J.C., D.M.D., G.D., D.K., K.L., D.R., P.S., J.N.S., G.A.W., S.M.W., C.B.B.). The project lead (D.D.O.) alongside the multidisciplinary steering committee members (C.A.B., J.C., D.M.D., G.D., D.K., K.L., D.R., P.S., J.N.S., G.A.W., S.M.W., and C.B.B.) and key partners (E.F., K.K., N.K., N.R., T.R., C.S., S.S., K.T., D.D.R., and S.W.T.) involved in co-dissemination of project findings in peer-reviewed publications, conferences, and with patient organizations.

**Table 2:** Participant demographic characteristics for each phase of the priority-setting process.

	Phase; Number (%) of Participants		
Characteristic	1: International Survey *n* = 74	3: Interim Prioritization *n* = 58	4: Priority-Setting Workshop *n* = 12
Key partner group			
People with lived experience	39 (53)	22 (38)	4 (33)
<18 years old	16 (41)	8 (36)	1 (25)
≥18 years old with childhood-onset	23 (59)	14 (64)	3 (75)
Family members/caregivers	18 (24)	21 (36)	3 (25)
Parent or primary caregiver	17 (94)	20 (95)	3 (100)
Other family member	1 (6)	1 (5)	0
Clinicians	17 (23)	15 (26)	5 (42)
Doctor	15 (88)	13 (87)	5 (100)
Anesthesia/pain medicine	11 (73)	9 (69)	3 (60)
Dermatology	1 (7)	0	1 (20)
Neurology	0	1 (8)	0
Rheumatology	3 (20)	3 (23)	1 (20)
Nurse	2 (12)	2 (13)	0
Location			
Canada	8 (11)	6 (10)	0
United States of America	53 (72)	38 (66)	8 (66)
United Kingdom	7 (9)	5 (9)	2 (17)
Australia	1 (1)	1 (2)	0
Other	5 (7)	8 (14)	2 (17)
Residence			
Urban	24 (32)	13 (22)	6 (50)
Suburban	43 (58)	33 (57)	4 (33)
Rural	7 (9)	12 (21)	2 (17)
Age, years			
Mean ± SD	36.3 ± 17.7	41.3 ± 16.7	42.2 ± 14.9
Range	8–73	12–71	22.6–63.5
Gender			
Male	24 (32)	16 (28)	0
Female	47 (64)	42 (72)	12 (100)
Prefer not to say	0	0	0
Prefer to self-describe	3 (4)	0	0
Ethnicity			
Indigenous, American Indian, or Alaska Native	1 (1)	2 (3)	0
Asian	7 (9)	5 (9)	2 (17)
Black or African American	0	0	0
Hispanic or Latino	1 (1)	1 (2)	0
Native Hawaiian or Other Pacific Islander	0	0	0
White	63 (85)	48 (83)	10 (83)
Interracial	2 (3)	1 (2)	0
Prefer not to say	0	1 (2)	0
**For patients/family members only**	*n* = 57	*n* = 43	*n* = 7
Years you (or family member) have lived with erythromelalgia			
Mean ± SD	15.4 ± 14.7	14.5 ± 11.4	13.4 ± 6.3
Range	1–58	2–50	4–25
Location of erythromelalgia care			
Specialty pediatric chronic pain clinic	8 (14)	15 (35)	5 (71)
Pediatric outpatient clinic	12 (21)	7 (16)	0
Family doctor/pediatrician	20 (35)	8 (19)	0
Other	21 (37)	10 (23)	2 (29)
None	14 (25)	10 (23)	0
**For healthcare providers/clinicians only**	*n* = 17	*n* = 15	*n* = 5
Years you have worked in pediatric erythromelalgia care			
Mean ± SD	17.8 ± 7.8	19.7 ± 10	13.4 ± 3.1
Range	8–30	8–40	10–18
Location of erythromelalgia care practice			
Specialty pediatric chronic pain clinic	13 (76)	11 (73)	4 (80)
Pediatric outpatient clinic	5 (29)	4 (27)	1 (20)
Family doctor/pediatrician	0	0	0
Other	0	0	0

Data presented as N (%), unless specified otherwise. SD: standard deviation.

## Data Availability

Data from phase 1 of the PSP may contain identifiable information due to personal experiences reported and therefore will not be made available. The REDCap surveys used for the PSP will be available from the corresponding author, upon reasonable request.

## Supplementary Materials

The following supporting information can be downloaded at https://www.mdpi.com/article/10.3390/children12111477/s1, Table S1: Verified research questions (N = 18); Table S2: Research questions for the interim prioritization; Table S3: Evaluation of patient engagement after the priority-setting workshop.

## Abbreviations

The following abbreviations are used in this manuscript:

PWLE	People with lived experience
PSP	Priority-setting process
PED-EMERGE	Multicenter PEDiatric ErythoMElalgia Registry Gathering multidisciplinary Experts

## References

[1] Ocay D.D., Maloney M.G., D’Souza G., Brownstein C.A., Clinch J., Davis D.M., De Ranieri D., Donado C., Halpin M., Kattail D. (2025). Pediatric erythromelalgia from multidisciplinary perspectives: A scoping review. Pediatr. Res..

[2] Marzban S., Najafi M., Agolli A., Ashrafi E. (2022). Impact of Patient Engagement on Healthcare Quality: A Scoping Review. J. Patient Exp..

[3] Katirai A., Kogetsu A., Kato K., Yamamoto B. (2022). Patient involvement in priority-setting for medical research: A mini review of initiatives in the rare disease field. Front. Public Health.

[4] The James Lind Alliance (2021). The James Lind Alliance Guidebook Version 10.

[5] Manafo E., Petermann L., Vandall-Walker V., Mason-Lai P. (2018). Patient and public engagement in priority setting: A systematic rapid review of the literature. PLoS ONE.

[6] Birnie K.A., Dib K., Ouellette C., Dib M.A., Nelson K., Pahtayken D., Baerg K., Chorney J., Forgeron P., Lamontagne C. (2019). Partnering for Pain: A Priority Setting Partnership to identify patient-oriented research priorities for pediatric chronic pain in Canada. CMAJ Open.

[7] Boney O., Bell M., Bell N., Conquest A., Cumbers M., Drake S., Galsworthy M., Gath J., Grocott M.P.W., Harris E. (2015). Identifying research priorities in anaesthesia and perioperative care: Final report of the joint National Institute of Academic Anaesthesia/James Lind Alliance Research Priority Setting Partnership. BMJ Open.

[8] Tutelman P.R., Thurston C., Rader T., Henry B., Ranger T., Abdelaal M., Blue M., Buckland T.W., Del Gobbo S., Dobson L. (2024). Establishing the Top 10 Research Priorities for Adolescent and Young Adult (AYA) Cancer in Canada: A Protocol for a James Lind Alliance Priority Setting Partnership. Curr. Oncol..

[9] Harris P.A., Taylor R., Minor B.L., Elliott V., Fernandez M., O’Neal L., McLeod L., Delacqua G., Delacqua F., Kirby J. (2019). The REDCap consortium: Building an international community of software platform partners. J. Biomed. Inform..

[10] Harris P.A., Taylor R., Thielke R., Payne J., Gonzalez N., Conde J.G. (2009). Research electronic data capture (REDCap)—A metadata-driven methodology and workflow process for providing translational research informatics support. J. Biomed. Inform..

[11] Abelson J., Li K., Wilson G., Shields K., Schneider C., Boesveld S. (2016). Supporting quality public and patient engagement in health system organizations: Development and usability testing of the Public and Patient Engagement Evaluation Tool. Health Expect..

[12] Abelson J., Tripp L., Kandasamy S., Burrows K., the PPEET Implementation Study Team (2019). Supporting the evaluation of public and patient engagement in health system organizations: Results from an implementation research study. Health Expect..

[13] Arthur L., Keen K., Verriotis M., Peters J., Kelly A., Howard R.F., Dib-Hajj S.D., Waxman S.G., Walker S.M. (2019). Pediatric Erythromelalgia and SCN9A Mutations: Systematic Review and Single-Center Case Series. J. Pediatr..

[14] Unniyampurath U., Pilankatta R., Krishnan M.N. (2016). RNA Interference in the Age of CRISPR: Will CRISPR Interfere with RNAi?. Int. J. Mol. Sci..

[15] Jha S.K., Karna B., Goodman M.B. (2025). Erythromelalgia. StatPearls.

[16] Xue Y., Chidiac C., Herault Y., Gaveriaux-Ruff C. (2021). Pain behavior in *SCN9A* (Nav1.7) and *SCN10A* (Nav1.8) mutant rodent models. Neurosci. Lett..

[17] Singh S., Chauhan S., Zeltser R. (2025). Mexiletine. StatPearls.

[18] Poulin P., Shergill Y., Romanow H., Busse J.W., Chambers C.T., Cooper L., Forgeron P.A., Harper A.O., Hudspith M., Iorio A. (2018). Researching what matters to improve chronic pain care in Canada: A priority-setting partnership process to support patient-oriented research. Can. J. Pain.

[19] Dormer A., Narayanan M., Schentag J., Achinko D., Norman E., Kerrigan J., Jay G., Heydorn W. (2023). A Review of the Therapeutic Targeting of SCN9A and Nav1.7 for Pain Relief in Current Human Clinical Trials. J. Pain Res..

[20] Magalhaes M. (2022). Should rare diseases get special treatment?. J. Med. Ethics.

