# Supplementary material for: Patient-Oriented Priorities for Pediatric Erythromelalgia: A Priority-Setting Process
Source: Children (Basel). 2025 Nov 2;12(11):1477. doi: 10.3390/children12111477 (PMC12650981; doi:10.3390/children12111477)
Supplement: Supplementary file 1 [file children-12-01477-s001.zip › children-3924051-supplementary.pdf]

SUPPLEMENTARY MATERIAL

Supplementary Table S1. Verified research questions (N = 18).

**Research question**	**Answer**
Can primary erythromelalgia be explained by SCN9A variants?	The recognition that some cases of familial erythromelalgia are linked to dominant gain-of-function mutations of the *SCN9A* gene led to the introduction of the term "inherited" primary erythromelalgia referring to patients with a confirmed genetic variant [13].
Can youth with erythromelalgia do physical activity/sports?	Our team's scoping review of the existing literature on youth with erythromelalgia revealed that although some cases may participate in physical activity, symptoms lead to limited physical activity.
Can youth with erythromelalgia have children in the future?	Yes, familial erythromelalgia cases have been published.
How are RNA interference (RNAi) techniques being utilized in SCN9A research, and what challenges have been encountered?	Although RNA interference is an important transient gene expression repression approach with various applications in the research on gene–phenotype relationships in mammalian systems, other gene editing techniques with more research and clinical advantages are now available, such as the CRISPR/Cas9 system [14].
How can youth with erythromelalgia access genetic screening?	This is, case by case, dependent on whether genetic screening is available at the patient's local institution or nearby institutions.
How can youth with erythromelalgia gain access to newer medications or trials, when they do not respond to traditional medications?	Clinical trials on erythromelalgia can be found on clinicaltrials.gov.
Is erythromelalgia hereditary?	Erythromelalgia may be idiopathic or inherited. The inherited form of erythromelalgia is an autosomal dominant neuropathy caused by a gain-of-function mutation in the SCN9A, SCN10A, and SCN11A genes which encodes the alpha subunit of the voltage-gated $Na_v1.7$, $Na_v1.8$, and $Na_v1.9$ sodium channels, respectively [15].
Is there a topical gel that can help reduce pain?<br><br>Are there any new options for cooling gels or creams?	A scoping review of the existing literature on youth with erythromelalgia revealed various combinations of pharmacological and non-pharmacological treatments and related responses reported across most studies, including topical gels [1].<br><br>Although there may be new options for cooling gels or creams, these may not be specific to erythromelalgia. Their clinical effectiveness in patients with erythromelalgia warrants study.
What are the latest findings regarding the role of the *SCN9A* gene in pain perception?	Recurrent gain-of-function mutations in this gene lead to an increase in cellular sodium influx, resulting in an increase in pain signal transmission that leads to erythromelalgia signs and symptoms. On the other hand, loss-of-function mutations in this gene decrease sensitivity to various pain stimuli. Future knowledge obtained from preclinical models with these mutations will allow us to understand how these mutations affect pain [16].
What are the long-term effects of mexiletine?	Mexiletine is primarily used to suppress ventricular arrhythmias and has shown a role in cases of youth of erythromelalgia, as revealed in a scoping review of the existing literature [1].<br><br>The adverse effect profile of mexiletine is extensive and therefore limits its use. It affects nearly every organ system: primarily the cardiovascular and central nervous systems but also the gastrointestinal, musculoskeletal, and dermatologic systems [17].
What are the most promising therapeutic approaches targeting the SCN9A gene currently being investigated?<br><br>What are the key challenges in translating SCN9A research findings into clinical applications?<br><br>Are there options for genetic therapies?	When looking at past and present human clinical trials targeting SCN9A and $Na_v1.7$, small molecules seem not to be very effective in the management of pain (clinicaltrials.gov).<br><br>Gene therapy, such as using the CRISPR/Cas9 system, could be a targeted way of engaging SCN9A therapeutically. However, preliminary results have shown challenges associated with adenovirus vectors delivering the CRISPR/Cas9 system therapeutic targeted intervention for the treatment of chronic pain via the downregulation of SCN9A. DNA plasmid vectors may be used as a more advantageous alternative. Nevertheless, further studies on the development of treatments targeting *SCN9A* are warranted [19].

What educational resources on erythromelalgia are available for patients, families, and clinicians?	The Erythromelalgia Association created and released "A Patient's Guide to Erythromelalgia" in Spring 2016. This open access publication is a comprehensive resource covering all aspects of the disease, from symptoms and diagnosis to treatment and daily coping tips. It is a unique tool that those with erythromelalgia (and their families and healthcare providers) should have at their fingertips as they strive to improve their lives with regard to EM (burningfeet.org/resources/patients-guide/).
What type of treatments are available to youth with erythromelalgia?<br><br>How have youth with erythromelalgia responded to medication?	A scoping review of the existing literature on youth with erythromelalgia revealed various combinations of pharmacological and non-pharmacological treatments and related responses reported across most studies. The most common pharmacological classes included adrenergic agonists, antihistamines, beta-blockers, calcium channel blockers, corticosteroids, cyclooxygenase inhibitors, opioid receptor agonists, sodium channel blockers, and selective serotonin/serotonin-norepinephrine reuptake inhibitors [1].
Would genetic testing for erythromelalgia be possible prior to IVF transfer?	Preimplantation genetic testing (PGT) examines embryos during in vitro fertilization before the possible transfer to a woman's uterus for a range of genetic problems that can cause implantation failure, miscarriage, and birth defects in a resulting child. The use of PGT to test for *SCN9A* may be feasible on a case-by-case basis. Please refer to your primary care physician and/or obstetrician gynecologist on this possibility.<br><br>It is important to note that although the *SCN9A* gene mutation has been linked to primary inherited erythromelalgia, familial cases without a confirmed *SCN9A* variant have been published.

PMID: PubMed ID

Supplementary Table S2. Research questions for the interim prioritization.

Research question chosen as their top 10, N (%)	All (*N* = 58)	PWLE (*n* = 22)	Caregivers (*n* = 21)	Clinicians (*n* = 15)
**ACCESS:** How can youth with erythromelalgia access multidisciplinary experts/management (including pain physicians, neurologists, rheumatologists, dermatologists, etc.)?	31 (53)	10 (45)	14 (67)	7 (47)
**ACCESS:** What strategies (e.g., social media, flyers, etc.) are effective to increase access to care for youth with erythromelalgia?	4 (7)	2 (9)	1 (5)	1 (7)
**GENETICS:** What are predisposing genes other than SCN9A to erythromelalgia?	24 (41)	12 (55)	5 (24)	7 (47)
**GENETICS:** What are the genetic markers for erythromelalgia and the likelihood that a child will develop erythromelalgia if they have that particular gene?	21 (36)	7 (32)	8 (38)	6 (40)
**GENETICS:** What is the most important genetic mutation for erythromelalgia?	9 (16)	3 (14)	5 (24)	1 (7)
**SUPPORT:** How can we get better support from schools for youth with erythromelalgia (e.g., presentations, information sheets)?	18 (31)	6 (27)	8 (38)	4 (27)
**SUPPORT:** How can youth with erythromelalgia get more support from the work environment (e.g., information sheets, speaking with colleagues, accommodations)?	9 (16)	5 (23)	4 (19)	0 (0)
**SUPPORT:** How can youth with erythromelalgia get support from family and friends?	6 (10)	4 (18)	2 (10)	0 (0)
**SUPPORT:** How can youth with erythromelalgia help others understand their perspective?	12 (21)	6 (27)	5 (24)	1 (7)
**SUPPORT:** Which strategies (e.g., support groups, social media) are the most effective to increase support for youth with erythromelalgia?	7 (12)	4 (18)	2 (10)	1 (7)
**KNOWLEDGE:** How can we increase the quantity and quality of evidence to guide treatment/management?	28 (48)	10 (45)	8 (38)	10 (67)
**KNOWLEDGE:** How can we increase awareness on pediatric erythromelalgia for the general population?	12 (21)	5 (23)	7 (33)	0 (0)
**KNOWLEDGE:** What are the different (pheno)types of erythromelalgia and their prognosis?	19 (33)	6 (27)	4 (19)	9 (60)
**KNOWLEDGE:** What are the pathophysiological mechanisms underlying pediatric erythromelalgia?	17 (29)	5 (23)	5 (24)	7 (47)
**KNOWLEDGE:** What is the association between erythromelalgia and autoimmunity?	17 (29)	7 (32)	7 (33)	3 (20)
**KNOWLEDGE:** What is the association between erythromelalgia and hormones/puberty?	10 (17)	4 (18)	5 (24)	1 (7)
**KNOWLEDGE:** What is the association between erythromelalgia and nutrition?	5 (9)	1 (5)	4 (19)	0 (0)
**KNOWLEDGE:** What is the association between erythromelalgia and weight gain?	1 (2)	1 (5)	0 (0)	0 (0)
**KNOWLEDGE:** What is the perspective of youth with erythromelalgia?	5 (9)	3 (14)	0 (0)	2 (13)
**KNOWLEDGE:** What is the psychosocial impact of erythromelalgia in youth?	4 (7)	1 (5)	3 (14)	0 (0)
**KNOWLEDGE:** What triggers flare-ups in youth with erythromelalgia?	13 (22)	3 (14)	5 (24)	5 (33)
**KNOWLEDGE:** Which strategies (e.g., continued education, flyers) are the most effective to educate more doctors about erythromelalgia for awareness and earlier diagnosis?	11 (19)	4 (18)	6 (29)	1 (7)
**KNOWLEDGE:** Which strategies (e.g., standard protocols/checklists) are the most effective to enhance the diagnostic process for youth with erythromelalgia?	8 (14)	1 (5)	2 (10)	5 (33)
**ACTIVITY:** What strategies are effective in managing flare-ups during physical activity?	20 (34)	8 (36)	9 (43)	3 (20)
**ACTIVITY:** What strategies are effective to allow youth with erythromelalgia to participate in physical activity/sports?	10 (17)	2 (9)	4 (19)	4 (27)
**LIFE:** What strategies are effective for maintaining a good quality of life (e.g., participating in activities, good peer relationships, self-esteem, etc.) in youth with erythromelalgia?	22 (38)	8 (36)	11 (52)	3 (20)
**LIFE:** What strategies (e.g., support groups, talking to schools) are effective to increase social activity for youth with erythromelalgia?	4 (7)	1 (5)	3 (14)	0 (0)
**SCHOOL:** What strategies are effective to increase school performance (e.g., attendance, academic performance, participation) in youth with erythromelalgia?	8 (14)	4 (18)	4 (19)	0 (0)
**SLEEP:** What strategies (e.g., coping mechanisms) are effective to increase sleep quality for youth with erythromelalgia?	16 (28)	9 (41)	7 (33)	0 (0)
**ADULTHOOD:** How can we provide continued care for youth with erythromelalgia?	8 (14)	4 (18)	2 (10)	2 (13)
**ADULTHOOD:** How can youth with erythromelalgia better navigate future romantic relationships?	2 (3)	2 (9)	0 (0)	0 (0)
**ADULTHOOD:** What are the long-term effects of taking medication for youth with erythromelalgia?	7 (12)	2 (9)	5 (24)	0 (0)
**ADULTHOOD:** What is the long-term effect of erythromelalgia in youth ("effect" refers to the result of having erythromelalgia during childhood, as an adult in the future)?	9 (16)	2 (9)	5 (24)	2 (13)

**ADULTHOOD:** What is the long-term trajectory of erythromelalgia in youth ("trajectory" refers to whether the condition will change over time)?	12 (21)	3 (14)	1 (5)	8 (53)
**ADULTHOOD:** What jobs are best for youth with erythromelalgia?	3 (5)	3 (14)	0 (0)	0 (0)
**TREATMENT:** How can we provide targeted/personalized treatment of youth with erythromelalgia?	24 (41)	10 (45)	5 (24)	9 (60)
**TREATMENT:** How effective are anticonvulsants (e.g., carbamazepine) for youth with erythromelalgia?	6 (10)	2 (9)	1 (5)	3 (20)
**TREATMENT:** How effective are gabapentinoids (e.g., pregabalin, Lyrica) combined with antidepressants (e.g., amitriptyline) for youth with erythromelalgia?	12 (21)	6 (27)	2 (10)	4 (27)
**TREATMENT:** How effective is avoiding triggers for youth with erythromelalgia?	0 (0)	0 (0)	0 (0)	0 (0)
**TREATMENT:** Which approaches are the most effective for preventing flare-ups in youth with erythromelalgia?	12 (21)	4 (18)	4 (19)	4 (27)
**TREATMENT:** Which approaches are the most effective for treating symptoms in youth with erythromelalgia?	15 (26)	4 (18)	5 (24)	6 (40)
**TREATMENT:** Which approaches are the most effective for treating itchiness in youth with erythromelalgia?	3 (5)	0 (0)	3 (14)	0 (0)
**TREATMENT:** Which approaches are the most effective for treating pain in youth with erythromelalgia?	13 (22)	4 (18)	4 (19)	5 (33)
**TREATMENT:** What are the guidelines for treating erythromelalgia?	7 (12)	2 (9)	2 (10)	3 (20)
**TREATMENT:** What channel blockers (type of medication) have shown to be most effective for sodium ion channels, particularly Nav1.7?	10 (17)	6 (27)	1 (5)	3 (20)
**TREATMENT:** What psychological strategies are effective in youth with erythromelalgia?	10 (17)	3 (14)	4 (19)	3 (20)
**TREATMENT:** What strategies are effective for the comorbidities (i.e., other health problems) accompanying erythromelalgia in youth?	6 (10)	2 (9)	3 (14)	1 (7)
**TREATMENT:** What type of medication provides minimal side effects to youth with erythromelalgia?	7 (12)	4 (18)	2 (10)	1 (7)
**TREATMENT:** Which coping strategies (e.g., breathing, cognitive behavioral therapy) are the most effective for youth with erythromelalgia?	5 (9)	2 (9)	2 (10)	1 (7)
**TREATMENT:** Which non-pharmacological strategies (e.g., physical therapy, psychological therapy) are effective for youth with erythromelalgia?	19 (33)	4 (18)	4 (19)	11 (73)

Cells highlighted in green represent questions aggregated into the top 25 for each partner group from the interim prioritization. Cells highlighted in blue represent the questions carried forward to the final workshop.
PWLE: people with lived experience.

Supplementary Table S3. Evaluation of patient engagement after the priority-setting workshop.

Item	Mean score ± SD (range)
**Communication and Supports for Participation**	
I had a clear understanding of the purpose of the Pediatric Erythromelalgia Priority-Setting Process.	4.7 ± 0.5 (4 - 5)
The supports I needed to participate were available (e.g., travel, childcare, etc.).	4.4 ± 0.8 (3 - 5)
I had enough information to contribute to the topic being discussed.	4.8 ± 0.4 (4 - 5)
Sharing Views and Perspectives	
I was able to express my views freely.	4.6 ± 0.9 (2 - 5)
I feel that my views were heard.	4.7 ± 0.7 (3 - 5)
A wide range of views on the topics discussed was shared.	4.7 ± 0.5 (4 - 5)
The individuals participating in the Pediatric Erythromelalgia Priority-Setting Process represented a broad range of perspectives on the topic.	4.7 ± 0.5 (4 - 5)
Impacts and Influence of the Engagement Initiative	
I think that the Pediatric Erythromelalgia Priority-Setting Process achieved its objectives.	4.6 ± 0.5 (4 - 5)
I am confident the input provided through this initiative will be used by the Pediatric Erythromelalgia Collaboration.	4.7 ± 0.5 (4 - 5)
I think the input provided through this activity will make a difference to the work of the organization.	4.5 ± 0.7 (3 - 5)
Final Thoughts	
As a result of my participation in the Pediatric Erythromelalgia Priority-Setting Process, I am better informed about erythromelalgia in children and teens and the research gaps.	3.8 ± 1.1 (2 - 5)
Overall, I was satisfied with this engagement initiative.	4.5 ± 0.7 (3 - 5)
This engagement initiative was a good use of my time.	4.7 ± 0.5 (4 - 5)

Open-ended questions	Emerging themes and representative comments
What else would you like us to know about how your participation in the Pediatric Erythromelalgia Priority-Setting Process was supported?<br><br>What else would you like us to know about how you were able to share your views?<br><br>What else would you like us to know about the influence you think the Pediatric Erythromelalgia Priority-Setting Process will have?<br><br>What were the strengths of the Pediatric Erythromelalgia Priority-Setting Process?<br><br>What could be improved about the Pediatric Erythromelalgia Priority-Setting Process?<br><br>What else would you like us to know about your experience with the Pediatric Erythromelalgia Priority-Setting Process?	Community engagement = 13<br>• The group worked together in a collaborative fashion and there was active listening…<br>• […] it was very validating to hear from other family members, patients and medical professionals to see how this impacts them. As a mom, it gave me hope for my child.<br>• Everyone was respectful and shared openly which made me feel more comfortable.<br>• It's great to know that our feedback will be used to influence the research taking place.<br><br>Organization of the process = 9<br>• The communication was very clear…<br>• The amount of steps and meetings were one of the strengths to me. I think it allowed all to share their opinion and it showed that the research team cared deeply about making sure all points were heard.<br><br>Future considerations = 2<br>• Giving patients and family members more background information about the process of research, and the significance of some of the more technical research statements, before the prioritization process.
